# Factors influencing successful establishment of exotic *Pinus radiata* seedlings with co-introduced *Lactarius deliciosus* or local ectomycorrhizal fungal communities

**DOI:** 10.3389/fmicb.2022.973483

**Published:** 2022-11-17

**Authors:** Ran Wang, Yanliang Wang, Alexis Guerin-Laguette, Peng Zhang, Carlos Colinas, Fuqiang Yu

**Affiliations:** ^1^Yunnan Key Laboratory for Fungal Diversity and Green Development, Germplasm Bank of Wild Species, Kunming Institute of Botany, Chinese Academy of Sciences, Kunming, China; ^2^Department of Crop and Forest Science, University of Lleida, Lleida, Spain; ^3^Mycotree C/-Southern Woods Nursery, Christchurch, New Zealand; ^4^Forest Sciences Center of Catalonia, Solsona, Spain

**Keywords:** exotic pine, extracellular enzyme, fungal community, *Lactarius*, rhizosphere, *Suillus*

## Abstract

An introduction of exotic or non-native trees may fail due to a lack of suitable fungal partners. We planted exotic *Pinus radiata* in Xifeng, Guizhou Southwest China. Strategies to introduce *P. radiata* seedlings either colonized with an ectomycorrhizal fungus (EcMF), *Lactarius deliciosus*, or expect them to form familiar/new associations with local EcMF in a new habitat were studied to know how *P. radiata* could be successfully established over a period of 2.5 years. Plant height and needle nutrient acquisition, the persistence of the co-introduced *L*. *deliciosus*, and fungal community composition in rhizosphere soil and root tips were analyzed. In addition, a greenhouse bioassay experiment of local soil to assess the differences in the EcMF community between exotic and native pine seedlings was also conducted. The current results demonstrated that *P. radiata* could establish in the Xifeng plantation with or without co-introduced *L*. *deliciosus.* The co-introduced *L*. *deliciosus* might be naturalized with *P. radiata* in the new area since it has been fruited for 2 years with high relative abundance in mycorrhizosphere soil. *L. deliciosus* pre-colonization significantly altered the mycorrhizosphere fungal composition and it had a positive correlation with nitrogen acquisition of *P. radiata.* Host identity had no effect on fungal composition since exotic *P. radiata* and native *P. massoniana* recruited similar local fungal communities in early establishment or in plantation. The cosmopolitan species *Suillus placidus*, with high relative abundance, formed a familiar association with *P. radiata*. The greenhouse bioassay experiment further showed that *Suillus* sp. contributed relatively higher total extracellular enzymes by forming ectomycorrhizas with *P. radiata* and the same type of ectomycorrhiza of *P. radiata* and *P. massoniana* showed different enzymatic functions. Our study indicated that exotic *P. radiata* could be a suitable tree capable to get established successfully in the Xifeng plantation either by interaction with the co-introduced *L. deliciosus* or with a local EcMF, but we should be cautious about large-scale planting of *P. radiata*. *L. deliciosus* persisted in plantation and more attention should be paid to local EcMF community changes induced by the introduced *L. deliciosus*.

## Introduction

A number of exotic tree species from the genera *Eucalyptus*, *Pinus*, and *Populus* have been introduced to China since the 1980s, which have become the dominant tree species in timber plantations (Song, 1983; Wang et al., 2006; Chen et al., 2018; Farooq et al., 2021). Among them, pine species are widely distributed in the Northern Hemisphere and play an important role in afforestation, due to their easy propagation, strong adaptability, short growth cycle, and high-quality wood (Song, 1983; Richardson et al., 2000; Wang et al., 2006). In nature, pine trees form obligate symbioses with a variety of ectomycorrhizal fungi (EcMF) which provide host plants with mineral nutrients, water, or defense against pathogens, whereas, plants supply EcMF with energy-rich carbon compounds in return (Allen, 1991; Liang et al., 2002; Smith and Read, 2008).

The successful establishment of exotic pines in non-native areas is dependent on various factors, such as pathway of introduction, soil properties, and local climate (Keane and Crawley, 2002; Pysek et al., 2011, 2015; Dodet and Collet, 2012; Dickie et al., 2017). Besides, some biotic factors such as pathogens, predators, and suitable mutualists also play important roles in the naturalization of introduced plants (Keane and Crawley, 2002; Pringle et al., 2009; Jurkien et al., 2020; Moyano et al., 2020; Ning et al., 2020). Ectomycorrhiza, a symbiosis formed between EcMF and plant roots, plays an essential role in the uptake of nutrients [e.g., nitrogen (N) and phosphorus (P)] and water from soil to support plant growth in forest ecosystems (Smith and Read, 2008). A variety of extracellular enzymes, including oxidase and hydrolase, are produced by EcMF to mobilize soil organic matter (Courty et al., 2005). Potential activities of extracellular enzymes involved in the degradation and nutrient release from soil organic matter have been used to address functional diversity among EcMF fungi *in situ* (Courty et al., 2005; Pysek et al., 2011). EcMF fungal species with specific enzyme activities are selected by hosts to fulfill their nutrient requirements.

Initial planting of pines failed in many parts of the Southern Hemisphere due to the lack of suitable EcMF (Marx, 1991; Pringle et al., 2009). It has been long recognized that the absence of coevolved EcMF in soils is a major obstacle to the successful establishment of introduced plants (Mikola, 1970; Poynton, 1979; Richardson et al., 2000; Pringle et al., 2009; Nuñez and Dickie, 2014). In general, there could be three strategies for exotic plants to overcome the detrimental loss of mutualistic symbionts: (1) interaction with EcMF co-introduced from the plant’s native habitats, (2) a familiar association with EcMF present in both native and new habitats, and (3) new associations with EcMF in a new habitat (Dickie et al., 2010, 2017).

By analyzing 42 independent global-scale datasets of EcMF communities associated with trees in the family Pinaceae and *Eucalyptus* species in the introduction areas, Vlk et al. (2020a) showed that the successful introduction of exotic Pinaceae relied on co-introduced EcMF, especially in areas where Pinaceae did not naturally exist. Co-introduced EcMF may preadapt to new areas as early successional fungi and then help exotic plants successfully establish in new areas (Vlk et al., 2020b). Many exotic tree species have been frequently transported to new areas as seedlings along with soil rather than specific inoculum from their native range, but the identities and distributions of EcMF co-introduced with Pinaceae species are poorly known (Mikola, 1970; Dickie et al., 2010; Hynson et al., 2013). Hayward et al. (2015) reported that the co-introduction of an EcMF was sufficient to successfully introduce *Pinus contorta* outside its natural distribution range or promote the expansion of its natural range. Ectomycorrhizal plants usually host highly diverse EcMF communities on their root system in their native range, thus whether a successful introduction is related to a few unique co-introduced EcMF remains questionable. On the other hand, co-introduced EcMF species may be replaced by local EcMF species or displace local EcMF (Richardson et al., 2000; Dell et al., 2002; Vellinga et al., 2009). Therefore, the ecology of an introduced EcMF at new sites needs to be studied during tree restoration.

Vlk et al. (2020a) summarized that familiar or new associations in new sites with native EcMF prevailed among exotic Pinaceae introduced to regions where other Pinaceae species occur naturally. The selection of high-efficiency partners in the local areas might be another important strategy for exotic pine establishment. Exotic plants may recruit different EcMF communities because of their phylogenetic distance (Trocha et al., 2012; Nguyen et al., 2016; Ning et al., 2021). In a bioassay experiment with natural soil cores from pine forests, Ning et al. (2020, 2021) documented that host identity was a key factor determining EcMF community assembled in plant early establishment and the shifts of EcMF community composition might happen to exotic pines for selecting beneficial mutualists against less beneficial counterparts (Hortal et al., 2017; Ning et al., 2019, 2021). In addition, the interaction between exotic pine species and local EcMF resulted in a higher capacity for organic nitrogen and phosphorus utilization than that of native pine species, which might indicate a host-specific advantage of plant-mycorrhiza symbiosis for nutrient uptake and cycling. A better understanding of such nutritional effects could provide insights into the mechanism of exotic plant establishment during ecosystem restoration.

*Pinus radiata* D. Don is a native species to the central coast of California (USA), which belongs to subsection *Australes* and section *Trifoliae* that have two to three needles per fascicle (Gernandt et al., 2005). *Pinus radiata* has the lowest mortality and best growth rate during early establishment, whereas other forest species are difficult to get established (Bi et al., 2003). Over the last 160 years, it has become one of the most widely planted exotic trees in the world, particularly in the Southern Hemisphere, mostly with the Mediterranean climate (Lewis and Ferguson, 1993; Lavery and Mead, 1998). In Australia, Chile, New Zealand, and South Africa, *P. radiata* is a mainstay of the forest economy serving domestic markets and generating income from exports (Lewis and Ferguson, 1993; Lavery and Mead, 1998; Toro and Gessel, 1999; Turner et al., 1999). Since 1990s, *P. radiata* has become the main planted tree species in New Zealand. One plantation of *P. radiata* has been established in Xifeng County, Guizhou Province, and the southwest of China since 2018. Seeds of *P. radiata* were from New Zealand.^1^ Mycorrhizal seedlings were obtained with a *Lactarius deliciosus* culture originally from fruiting bodies in New Zealand’s *P. radiata* plantations (Wang et al., 2002; Guerin-Laguette et al., 2014; Wang et al., 2019). There was no record of *Lactarius deliciosus* fruiting bodies in the area before the Xifeng plantation establishment. Our primary purpose was to cultivate *L. deliciosus* and reuse wasteland. In May 2018, mycorrhizal and non-inoculated seedlings of exotic *P. radiata*, and non-mycorrhizal seedlings of native *P*. *massoniana* Lamb, were respectively planted in Xifeng County, Guizhou Province.

In this present study, the following studies in the plantation were conducted: (a) seedlings’ height was measured every 6 months, (b) nutrient concentration in needles was analyzed after *L. deliciosus* fruiting, (c) the composition and diversity of the EcMF communities were analyzed through high-throughput sequencing, and (d) a greenhouse bioassay experiment was conducted by planting exotic *P. radiata* and native *P. massoniana* seedlings in soils collected from Xifeng and activity of extracellular enzymes related to C, N, and P acquisition were assayed in ectomycorrhizal root tips. Based on the above-mentioned analysis, we aimed to assess the effect of co-introduced *L. deliciosus* on the rhizosphere fungal community and exotic *P. radiata* establishment. Moreover, the impact of host identity on structure and function of fungal community was also examined. The following questions were addressed: (1) Are there differences in growth effect and nutrient acquisition between *P. radiata* with or without *L*. *deliciosus* and *P. massoniana* seedlings?, (2) Are there differences in diversity and relative abundance of fungal communities in rhizosphere soil of co-introduced *P. radiata* with or without *L*. *deliciosus*?, (3) What is the difference in the EcMF communities recruited by exotic *P. radiata* and native *P. massoniana* in their early establishment under greenhouse and field plantation?, and (4) Are there distinctive relationships in ectomycorrhizal enzyme activity and nutrient content of tissue between native and exotic pine seedlings? Answers to the above-mentioned questions would improve our understanding of how an introduced EcMF *L. deliciosus* or local EcMF could help an exotic *P. radiata* to be successfully established in a new habitat.

## Materials and methods

### Study sites

**FIGURE 1:**
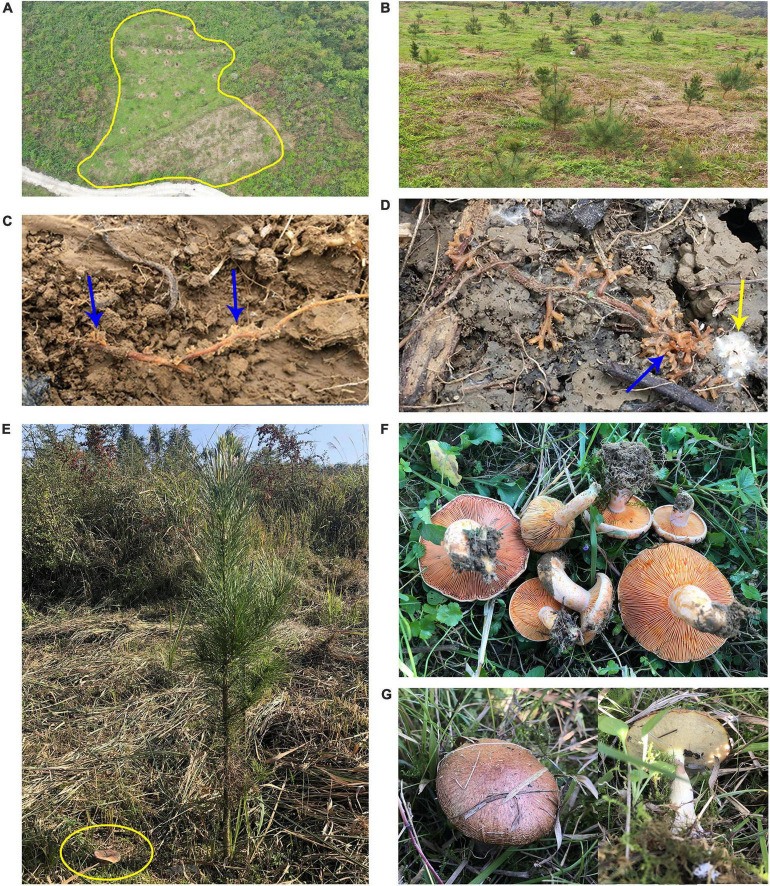
Plantation in Xifeng and mushroom production in plantation in November 2020. **(A)** Study site (yellow line). **(B)** Trees in the plantation 2.5 years after planting, including *Pinus radiata* + *Lactarius deliciosus* (PR + LD), *P. radiata* (PR) and *P. massoniana* (PM). **(C)** Ectomycorrhizas of PR + LD (blue arrows showing ECM tips). **(D)** *Suillus* colonization to roots of PR + LD (yellow arrow). **(E)** *L. deliciosus* fruiting bodies (yellow circle) occurred about one meter from the PR + LD tree. **(F)** *L*. *deliciosus* fruiting bodies from four PR + LD trees. **(G)** *Suillus* sp. fruiting bodies occurred near the PR tree.

The plantation (0.67 ha, Figures 1A,B) is located in Xifeng County (27° 21′ N, 106°41′ E, alt. 1,213 m above the sea level), Guizhou province, and southwest China, where the climate belongs to subtropical monsoon humid climate and the annual rainfall is ∼1,200 mm. The plantation area was an abandoned farmland where shrubs and weeds (dominated by *Pyracantha* sp. and Fabaceae) had grown for more than 5 years. The native *Pinus massoniana* (PM) forests are 1,000 m from the plantation. Soil’s physical and chemical properties were tested before the plantation (Table 1). *Pinus radiata*-*Lactarius deliciosus* (PR + LD) seedlings were obtained by inoculating vegetative inoculum in the greenhouse in October 2017, according to the method of Wang et al. (2019). Isolates of LD were originated from good quality fruiting bodies collected from areas in northern Europe which had a similar climate to the area in New Zealand where it was planned to grow them.

**TABLE 1:** Soil physico-chemical characteristics in the Xifeng plantation.

^1^Properties	Xifeng (*n* = 10)
pH (H_2_O)	6.30 ± 0.10
Sand (%)	24.00 ± 1.40
Silt (%)	26.00 ± 1.10
Clay (%)	50.00 ± 1.00
C (g kg^–1^)	15.80 ± 3.30
Total N (g kg^–1^)	1.45 ± 0.20
Total P (mg kg^–1^)	243.50 ± 38.90
Total K (mg kg^–1^)	9362.00 ± 426.40
Total Ca (mg kg^–1^)	1458.00 ± 128.00

^1^Values represent means ± standard errors.

Before plantation establishment, all seedlings were checked morphologically under a stereomicroscope (Leica Microsystems GMBH, Germany) to confirm mycorrhization. Ectomycorrhizal root tips from PR + LD seedlings and root tips from PR and PM seedlings were randomly selected for DNA extraction, then DNA extracts were checked the quality and quantity. The ITS1 region was amplified and sequenced on an Illumina MiSeq platform (see Supplementary Method 1 for fungal community sequencing, Supplementary Figure 1). In May 2018, a total of 34 PR + LD, 10 PR, and 24 PM seedlings were randomly planted in the Xifeng plantation. Planting holes (approximately 30 cm × 30 cm × 30 cm) were moistened with water for 24 h in order to soften the soil. Seedlings were planted in each block which was spaced at 4 m × 4 m. Because the plot is irregular and rugged with rocks, some trees have different spacings (Supplementary Figure 2). In November 2020, LD fruiting bodies have been observed in the plantation with the mycorrhizal, not with the non-mycorrhizal seedlings (Figures 1E,F). Results of simple sequence repeat confirmed that fruiting bodies were from co-introduced LD isolate (unpublished data).

### Sampling procedure in plantation

Seedlings’ height was measured every 6 months from November 2018 to May 2021 (T1∼T6). The survival rate of seedlings was also recorded 1 year after planting. In March 2021, five plants of each tree species were randomly selected and the soil layer was lightly excavated at 0.5 and 1.0 m from the trunk to observe mycorrhizae or fine roots. The average rainfall was 0.8 mm and the temperature in the day was 9∼16°C in March 2021. Soil samples were collected with a sterilized stainless steel spoon within 1.0 cm around mycorrhizae or roots. The corresponding mycorrhizae or roots were cut with a sterile scissor and the soil attached to the mycorrhizae or roots was gently shaken off and then collected. All samples were stored in sterile centrifuge tubes and put in foam boxes with ice packs to maintain the temperature at 2∼8°C, then brought back to the laboratory as soon as possible. Mycorrhizal tips and roots were gently washed with sterile water and then collected after the removal of soil debris. All samples were then used for Illumina MiSeq sequencing (see details in Supplementary Method 1). The raw sequence data have been deposited in the NCBI Sequence Read Archive database under the BioProject identifier PRJNA 795335.

Healthy mature pine needles from the center of branches in the center part of the sampling trees were sampled, and their fresh and dry weight (48 h at 65°C) was measured. The dried needles were then ground into fine powder and the content of carbon (C) and nitrogen (N) was determined by a Vario MAX CN instrument (Elementary Analyse system GmbH, Hanau, Germany). Total phosphorus (P), potassium (K), calcium (Ca), iron (Fe), and manganese (Mn) were determined with an inductively coupled plasma atomic-emission spectrometer (IRIS Advantage-ER; Thermo Jarrell Ash Corporation, Boston, MA, USA) by ICP-AES method.

### Bioassay experiment in greenhouse

#### Soil collection from plantation

Using a steel soil auger (10 cm diameter and ∼12 cm deep), we collected 20 soil cores in wastelands next to the Xifeng plantation in March 2021. The soil cores were kept intact and undisturbed. We put them in sealed ziplock bags and transported them with ice to maintain the temperature of 2∼8°C to the laboratory (Ning et al., 2020). Soils from 10 of the cores were placed into 688-ml plastic pots (top diameter 9.1 cm, bottom diameter 6.4 cm, and height 13.2 cm, S9, Xinguanghe horticulture, Zhejiang) which were pre-sterilized with sodium hypochlorite (2% available chlorine), while the other 10 soil cores were autoclaved for 3 h at 121°C to be used as control. The soil was sown with either PR seeds (Proseed, New Zealand) or native PM seeds (Guizhou, Duyun City). Before sowing, seeds were surface sterilized with 30% hydrogen peroxide for 10 min (PR seeds) or 5 min (PM seeds), then soaked in sterile water, and stored at 4°C for 3 weeks (Ning et al., 2020). After germination, eight seedlings were kept in each pot. Plants were grown in a randomized block design for pot arrangements in a greenhouse at the Kunming Institute of Botany (KIB). Growth conditions were natural day-length (12–14 h), e.g., 169 μmol^–2^s^–1^ inside the greenhouse in June and the average temperature in the day was 12–28°C.

#### Mycorrhizae formation and identification

After 3 and 6 months of seed germination (June and September 2021), three pine seedlings were harvested and then gently washed with tap water. The number of root tips and root tip area were estimated using W_IN_R_HIZO_ (Regent Instruments Canada Inc., Quebec, Canada). Types of root tips of ectomycorrhiza (ECM) were identified from morphological, anatomical (Agerer, 1987-2002, 1991), and molecular characteristics (Karen et al., 1997). The sorted homogeneous ECM morpho-anatomical types were counted. Ectomycorrhiza (ECM) colonization was calculated as the number of ECM root tips divided by the total number of root tips which include ECM root tips and non-ECM root tips (Gehring and Whitham, 1994).

#### Extracellular enzyme activity of ectomycorrhiza

The activity of cellobiohydrolase (C1, 3.2.1.91), β-glucosidase (GC, 3.2.1.21), β-glucuronidase (GD, 3.2.1.31), β-xylosidase (X, 3.2.1.37), laccase (LAC, 1.10.3.2), *N*-acetylglucosaminidase (NAG, 3.2.1.14), leucine amino peptidase (LEU, 3.4.11.1), and acid phosphatase (ACP, EC 3.1.3.2) was assessed with ectomycorrhizal tips in 96-well filter plates (Pritsch et al., 2011; Ning et al., 2020). Substrates, standards, and solutions were prepared according to protocols. Each of the three sampled root tips per morphotype and per sample was placed individually into 96-well filter plates. One column of the plate was left empty for the control of background fluorescence and another one for the calculation of a standard curve. Potential enzyme activity is subsequently expressed as a rate in pmol mm^–2^ min^–1^.

#### Plant harvest and tissue C, N, and P measurements

Three pine seedlings per pot were sampled and separated into shoot and root, dried for 48 h at 60°C, and then their biomasses were weighed. Next, dried tissues were ground into fine powder, and root and shoot C, N, and P were analyzed. Carbon (C) and nitrogen (N) were determined by a Vario MAX CN instrument (Elementar Analyse system GmbH, Hanau, Germany). Total phosphorus (P) was determined with an inductively coupled plasma atomic-emission spectrometer (IRIS Advantage-ER; Thermo Jarrell Ash Corporation, Franklin, MA, USA).

### Data analyses

Data (means ± SE, *n* = 6) from the plantation were statistically analyzed by *R* software version 3.2.3 (R Core Team, 2015). A one-way analysis of variance for independent samples was performed. Pearson correlation analysis (for all analyzed samples, *n* = 12) was used to examine the correlations between the relative abundance of EcMF OTUs and measured needle nutrient concentrations. Statistical significance for Pearson correlation was determined by pairwise two-sided comparisons. Fungal α-diversity was estimated by richness (Chao 1) and diversity (Simpson) indexes. Patterns in ECM fungal community composition were analyzed and visualized with principal coordinates analysis (PCoA) based on Bray–Curtis distance at the OTU level. The PERMANOVA analysis with Python’s scikit-bio package on Bray–Curtis dissimilarity matrices was performed to examine variation in the composition of EcMF with respect to the co-introduced LD and host interactions. The shared or unique fungal OTU numbers (among soil or roots of different hosts) were calculated based on the OTU abundance matrix using the nVennR package in R software. A significance α level of 0.05 was applied.

The relative contribution of each EcMF group to community enzyme activity in each pot was calculated using the following formula: Activity of EcMF group/total activity of EcMF community per seedling. Pearson correlation analysis was performed to examine the correlations between the enzyme activity and plant tissue parameters of biomass production and nutrient concentrations.

## Results

### In plantation

#### Growth effect of trees and needle nutrient concentration responses

We did not find any significant difference (*P* > 0.05) in plant height increment between PR + LD and PR but both were significantly higher than PM (*P* < 0.05) (Table 2 and Supplementary Figure 3). The Inoculation of PR trees showed no significant effects on needle C, N, P, K, Ca, Fe, and Mn concentrations, either (Table 3). Needle C (*P* < 0.01), P (*P* < 0.05), and Fe (*P* < 0.05) concentrations of PM were significantly higher than that of PR or PR + LD, but no significant differences are found in N, K, Ca, and Mn concentrations and water content. In addition, 1 year after planting, the mean survival rate was highest in PR + LD (94.1%), next was PR (90%), and lowest was PM (58.3%).

**TABLE 2:** Average plant height (cm) of trees in the Xifeng plantation every 6 months.

	^1^May-2018	November-2018	May-2019	November-2019	May-2020	November-2020	May-2021
PR + LD (*n* = 6)	18.08 ± 3.50a	45.80 ± 12.07a	76.80 ± 21.32a	103.80 ± 23.94a	140.00 ± 26.84a	162.80 ± 32.17a	203.4 ± 45.97a
PR (*n* = 6)	15.84 ± 3.42ab	33.2 ± 7.76a	56.6 ± 10.76a	85.4 ± 9.94a	120.6 ± 17.98a	147.2 ± 30.89a	171 ± 41.31a
PM (*n* = 6)	13.42 ± 1.53c	12.2 ± 1.30b	32.3 ± 11.17b	40.2 ± 8.98b	81.4 ± 15.95b	91.4 ± 16.82b	117.8 ± 12.64b

^1^Values represent means ± standard errors. Different letters indicate significant (*P* < 0.05) differences between individual means assessed by two-way factorial analysis of variance (ANOVA) followed by Least-Significant Difference testing.

**TABLE 3:** Needle nutrient concentrations of trees in the Xifeng plantation.

	^1^PR + LD (*n* = 6)	PR (*n* = 6)	PM (*n* = 6)
C (%)	49.72 (0.41) b	49.32 (0.41) b	50.22 (0.48) a
N (%)	1.42 (0.14)	1.36 (0.11)	1.56 (0.27)
Total P (mg g^–1^)	1.12 (0.05) b	1.02 (0.19) b	1.20 (0.08) a
Total K (mg g^–1^)	5.16 (0.82)	4.14 (1.39)	3.8 (0.77)
Total Ca (mg g^–1^)	2.11 (0.95)	2.28 (0.99)	1.38 (0.39)
Total Fe (mg g^–1^)	0.25 (0.07) b	0.28 (0.033) b	0.34 (0.03) a
Total Mn (mg g^–1^)	0.23 (0.03)	0.25 (0.05)	0.32 (0.15)

Data represents mean with the standard error in parentheses. ^1^Values represent means ± standard errors. Different letters indicate significant (*P* < 0.05) differences between individual means assessed by two-way factorial analysis of variance (ANOVA) followed by Least-Significant Difference testing.

#### Fungal community in mycorrhizosphere soil, rhizosphere soil, and roots

##### *Pinus radiata* + *Lactarius deliciosus* and *Pinus radiata*

**FIGURE 2:**
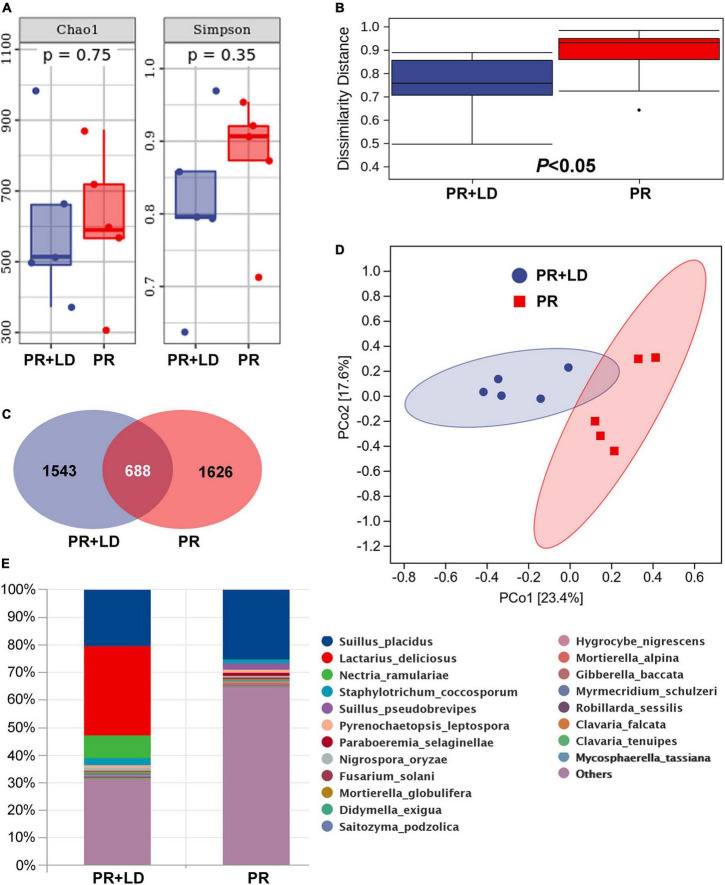
Mycorrhizosphere and rhizosphere fungal community of *Pinus radiata* + *Lactarius deliciosus* (PR + LD) and PR trees in the Xifeng plantation. **(A)** Chao1 and Simpson indexes. **(B)** Dissimilarity distance. **(C)** Venn figure showing shared and unique operational taxonomic units (OTUs) between samples of two trees. **(D)** Principal Coordinate analysis (PCoA). **(E)** Relative abundance of fungal composition at the species level.

In mycorrhizosphere and rhizosphere soil, fungal community analysis indicated significant differences in β-diversity (dissimilarity distance, Figure 2B) but not in Chao1 and Simpson index (α-diversity, Figure 2A), between PR + LD and PR trees in the Xifeng plantation. These two groups were clearly defined by the PCoA (Figure 2D). About 17.8% OTUs of 3,857 total OTUs were shared by both trees (Figure 2C). Fungal species with a relative abundance in the top 20 included EcMF (e.g., *Suillus*), plant pathogens (e.g., *Fusarium*, *Nectria*), saprophytic (e.g., *Hygrocybe*, *Clavaria*), and endophytic fungal species (e.g., *Nigrospora*) (Figure 2E). *Suillus* genus from EcMF was common and dominated in soil (20.4% of PR + LD, 27.4% of PR). Among the genus, *Suillus placidus* has the highest relative abundance and is significantly higher than other *Suillus* species. Based on Species Hypotheses (SH) in the UNITE database,^2^ OTU_3575 (*Suillus placidus*) was classified as cosmopolitan species and usually formed ectomycorrhizas with *Pinus* (Supplementary Table 1). A total of 86 detected OTUs from mycorrhizosphere and rhizosphere soil were EcMF species, but all of them were detected with low relative abundance except *Suillus* spp. (Figure 4).

**FIGURE 3:**
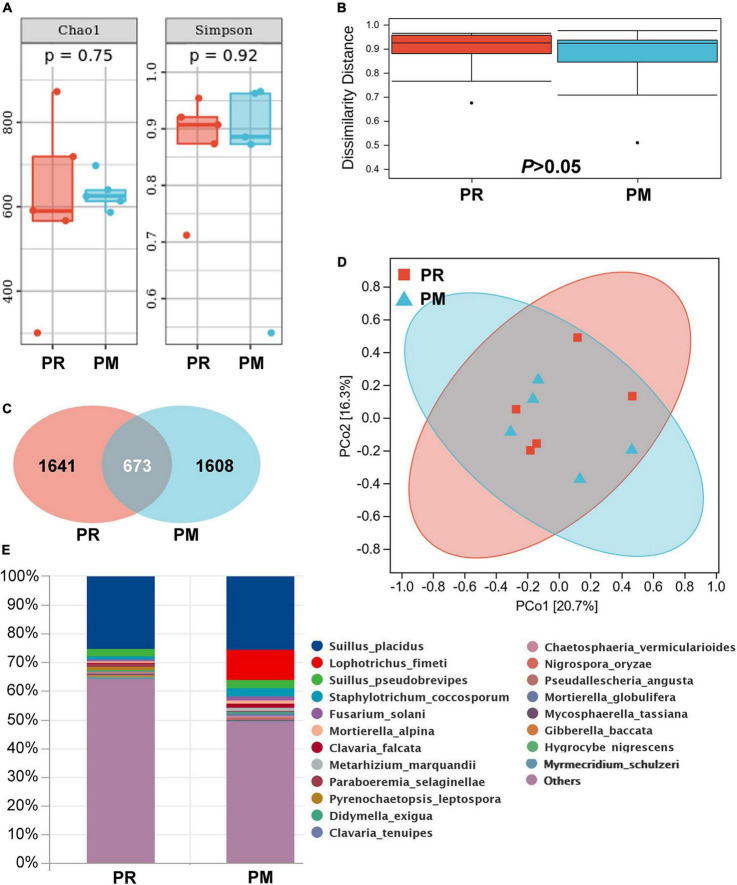
Rhizosphere fungal community of *Pinus radiata* (PR) and *Pinus massoniana* (PM) trees in the Xifeng plantation. **(A)** Chao1 and Simpson indexes. **(B)** Dissimilarity distance. **(C)** Venn figure showing shared and unique operational taxonomic units (OTUs) between samples of two trees. **(D)** Principal Coordinate analysis (PCoA). **(E)** Relative abundance of fungal composition at the species level.

**FIGURE 4:**
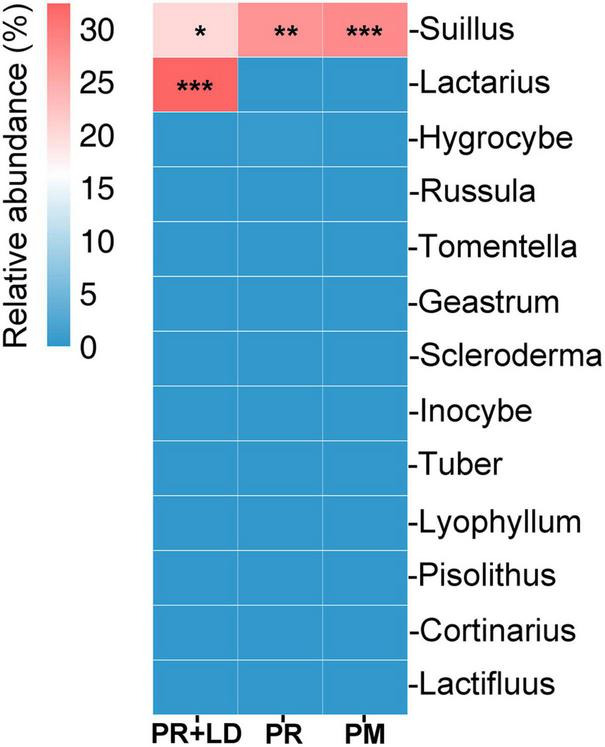
Heatmap of the relative abundance of ectomycorrhizal fungi operational taxonomic units (OTUs) at genus from mycorrhizosphere and rhizosphere soil of *Pinus radiata* + *Lactarius deliciosus* (PR + LD), PR and *Pinus massoniana* (PM) (agglomerated to genus). *P < 0.05; **P < 0.01; ***P < 0.001.

In the soil of PR + LD, *Lactarius deliciosus* (32.4%) was dominating but showed no significant difference with the relative abundance of *S. placidus* (*P* = 0.548). The Pearson correlation analysis revealed significant correlations between the relative abundance of *L. deliciosus* in mycorrhizosphere soil and nitrogen acquisition of needles of PR (*r* = 0.89, *P* = 0.018, *n* = 12). In ectomycorrhizal tips of PR + LD, fungal community analysis indicated that LD was dominating (94.5%) in the tips, while 5% of *S. placidus* (OTU_3575) was also colonizing the tips. In the plantation, mycorrhizal tips with *Suillus* were found beside mycorrhizal tips with *L. deliciosus* (Figure 1D). In root tips of PR, the relative abundance of *S. placidus* (OTU_3575) and *S. pseudobrevipes* (OTU_9078) was significantly greater than that of other EcMF.

##### *Pinus radiata* and *Pinus massoniana*

Fungal community analysis indicated no significant differences in α-diversity and β-diversity (Figures 3A,B,D) between PR and PM. A total of 3,922 OTUs were displayed and about 17.2% OTUs were shared by both trees (Figure 3C). *Suillus* genus was common in soil (28.2%) (Figure 3E) and root tips (33.5%) of PM, which included *Suillus* OTU_3575, OTU_5689, and OTU_9078. In the root tips of PR, *Suillus* included OTU_3575 and OTU_9078. The Pearson correlation analysis revealed significant correlations between the relative abundance of *Suillus* and manganese acquisition of needles in PM (*r* = 0.89, *P* = 0.044, *n* = 12). A total of 84 of the detected OTUs from the rhizosphere soil were EcMF species, but all of them were detected with low relative abundance except those in the genus *Suillus* (Figure 4).

### In greenhouse

#### Ectomycorrhizal fungi community composition in early establishment

**FIGURE 5:**
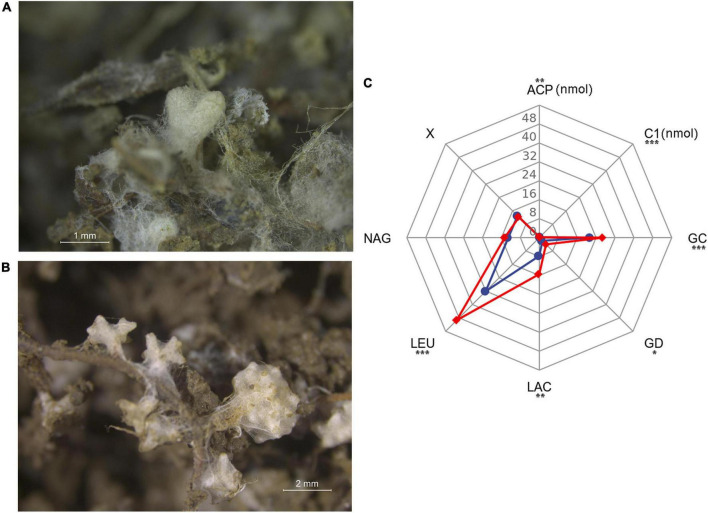
Bioassay experiment in greenhouse. **(A)** Mycorrhizal root tips of *Pinus radiata* (PR). **(B)** Mycorrhizal root tips of *Pinus massoniana* (PM). **(C)** Enzyme activity (pmol tip^–1^min^–1^/C1 and ACP: nmol tip^–1^ min^–1^) profiles of mycorrhizal root tips. PR seedlings-red line, PM seedlings-blue line. C1, cellobiohydrolase; GC, β-glucosidase; GD, β-glucuronidase; LAC, laccase; X, β-xylosidase; LEU, leucine aminopeptidase; NAG, *N*-acetylglucosaminidase; ACP, acid phosphatase. *P < 0.05; **P < 0.01; ***P < 0.001.

*Pinus radiata* and PM seeds germinated in the soil from the Xifeng plantation after 3 months and only two PM seedlings formed one type of mycorrhizae, and it was identified as *Suillus* sp. by molecular identification. After 6 months, PR and PM formed *Suillus*-like mycorrhizae (Figures 5A,B). The mycorrhizae samples were also identified as *Suillus* sp. (GenBank No: OM131553 and OM131554). Illumina sequencing detected OTUs as *Suillus placidus* (OTU_1487 and OTU_4508) (Supplementary Table 2). Ectomycorrhizal fungi (EcMF) colonization rates of the two hosts showed no significant difference (Table 4). No contamination of ectomycorrhizal fungi was found in control seedlings.

**TABLE 4:** Mean levels of seedling vigor status and tissue nutrient concentrations of seedlings in greenhouse bioassay experiment.

	^1^PR + *Suillus* (*n* = 15)	PM + *Suillus* (*n* = 15)	PR (*n* = 15)	PM (*n* = 15)
Shoot biomass (g)	0.48 (0.014)	0.19 (0.006)	0.44 (0.03)	0.18 (0.01)
Root biomass (g)	0.34 (0.004)	0.106 (0.009)	0.30 (0.02)	0.095 (0.07)
Root tip number	243.00 (7.52)	229.20 (11.69)	221.80 (7.19)	220.60 (11.41)
ECM colonization (%)	25.40 (3.26)	28.94 (1.89)	0.00	0.00
C (mg g^–1^)	489.78 (13.51)	469.19 (4.38)	483.53 (17.86)	465.02 (7.56)
N (mg g^–1^)	9.27 (0.51) b	12.54 (0.79) a	9.27 (0.71) b	10.1 (0.72) b
P (mg g^–1^)	0.72 (0.05) ab	0.82 (0.07) a	0.39 (0.01) b	0.34 (0.01) b

Data represents mean with the standard error in parentheses. ^1^Values represent means ± standard errors. Different letters indicate significant (*P* < 0.05) differences between individual means assessed by two-way factorial analysis of variance (ANOVA) followed by Least-Significant Difference testing.

#### Exoenzyme activities of ectomycorrhiza

Eight enzyme activities of tips indicated a clear separation of enzymatic functions associated with the two hosts (Figure 5C and Supplementary Table 3). *Suillus* sp. associated with PR seedlings had relatively higher C1, GC, GD, LAC, LEU, and ACP activities than those of PM seedlings. There were no significant differences of X and NAG activities between the two hosts. The total enzyme activity of PR was significantly greater than that of PM (*P* < 0.001).

#### The effect of exoenzyme activities on seedling nutrient acquisition

Extracellular enzyme activities showed significant effects on seedling biomass and tissue nutrient concentrations (Supplementary Table 4). The Pearson correlation analysis revealed a positive correlation between LAC, NAG, and ACP activity and shoot biomass of PR seedlings, and between LEU activity and root biomass of PR seedlings. P content of PR tissue was associated with GD enzyme activity. For PM seedlings, X enzyme activity had a positive correlation with C content.

## Discussion

In this study, we found no effects of the co-introduced *Lactarius deliciosus* on plant growth and nutrient acquisition of exotic PR in the Xifeng plantation. *Lactarius deliciosus* (LD) persisted in the plantation and was not replaced by local EcMF for the moment. The high relative abundance of LD had a positive correlation with nitrogen acquisition of PR. *Lactarius deliciosus* (LD) had no effects on rhizosphere fungal richness or diversity but significantly altered its fungal composition. Host identity had no effect on the fungal community of exotic PR pines and native PM pines by being colonized well by native EcMF *Suillus* in plantation or early establishment. However, the mycorrhizal symbiosis of PR had higher total EcMF enzymatic activity than PM pines and different enzymatic functions.

### Adopted strategy for establishment of exotic *Pinus radiata* in the Xifeng plantation

In the plantation, the co-introduced LD dominated in the mycorrhizosphere of PR + LD trees, and the high relative abundance of LD had a positive correlation with nitrogen acquisition of needles of PR. Lilleskov et al. (2011) showed that genera including *Thelephora*, *Laccaria*, and *Lactarius* exhibit a largely positive relationship with N availability. In addition, it was easy to find LD ectomycorrhizas around PR + LD trees (Figure 1C) and LD fruiting bodies were collected in 2020 (13 fruiting bodies) and 2021 (11 fruiting bodies). Whilst EcMF *Suillus* sp. formed a familiar association with exotic PR and the mycorrhizas contributed relatively higher extracellular enzymes than PM-*Suillus* mycorrhiza. No significant difference in plant growth and nutrient acquisition between PR + LD and PR indicated that PR could establish successfully with or without the co-introduced LD.

Vlk et al. (2020a) proposed that the share of strategies that alien Pinaceae species adopted to establish EcMF partnership in new areas differed greatly among biogeographic regions. Exotic Pinaceae were almost exclusively associated with co-introduced EcMF in areas without native Pinaceae (e.g., Africa, Oceania, and South America), whereas, the familiar or novel association with native EcMF prevailed in regions where other Pinaceae family members naturally occur. In Asia, only very few co-introduced EcMF associated with exotic Pinaceae are found in new areas where other Pinaceae species naturally occur. In our study, the Xifeng plantation is about 1 km away from native PM forests and it was used for planting crops for a long time. The soil spore banks may be limited, but we still found *Suillus* spp. with a high relative abundance in the soil, which is likely to come from extensive and long-lived soil spore banks of resistant propagules or native PM forests by spore dispersal (Peay et al., 2011). Glassman et al. (2015, 2016) demonstrated that *Suillus* is a dominant ECM component in the spore bank in soils in North America and may be extremely important in fungal colonization after large-scale disturbances, such as clear cuts and forest fires.

### Persistence of *Lactarius deliciosus* in the Xifeng plantation

*Lactarius deliciosus* (LD) persisted in mycorrhizosphere soil with a high relative abundance and LD fruiting bodies have been producing for 2 years in the Xifeng plantation. Although there was no effect of pre-inoculated LD on the growth and nutrients acquisition of PR in the plantation, LD with high relative abundance still showed a positive correlation of nitrogen acquisition of PR. An introduced species has the potential to reach any of the four stages: transport, establishment, spread, and impact (Lockwood et al., 2007). Vellinga et al. (2009) grouped fungal examples from the literature into five different outcomes: (1) fail to establish, (2) be replaced by local fungi, (3) persist with introduced trees but fail to grow with local hosts, (4) persist with introduced trees and spread to local hosts, or (5) fail to persist with introduced trees but nonetheless spread to local hosts. Our study so far demonstrated that LD may persist with the introduced PR and naturalize outside the native range.

This result is the first report on continuous basidiocarps of LD formation in China. The present work will contribute to establishing LD orchards in abandoned lands. Meanwhile, the cultivation of edible mycorrhizal fungi represents a novel farming activity that can satisfy an increasing demand for gourmet food and contribute to the conservation of natural ecosystems (Savoie and Largeteau, 2011). In addition, LD basidiocarp studies will provide information about changes in reproductive output, and will be a good indicator of belowground abundance.

### Co-introduced *Lactarius* altered mycorrhizosphere fungal communities

Co-introduced LD significantly altered the mycorrhizosphere fungal composition in the Xifeng plantation. Extraradical mycelium expansion of inoculated LD in the soil may compete for nutrients and produce secondary metabolites that affect the microbe community composition of the mycorrhizosphere (Fransson and Rosling, 2014). The composition of EcMF communities may largely affect ecosystem processes, such as carbon (Chapela et al., 2001) or nutrient cycling (Read and Perez-Moreno, 2003; Clemmensen et al., 2014). It is still needed to further verify whether the changes in fungal community composition influence nutrient cycling in local soils and nutrient uptake of exotic PR, as well as LD’s capacity, to expand into the native forests becoming an invasive species.

### Interaction effects between local ectomycorrhizal fungus communities and hosts

We found that *Suillus* spp. with a high relative abundance was detected in the mycorrhizosphere and rhizosphere soil in the Xifeng plantation. Fruiting bodies of *Suillus* were collected around PR trees (Figure 1G). *Suillus* or *Rhizopogon* species showed host-specificity to the Pinaceae family (Ashkannejhad and Horton, 2006; Collier and Bidartondo, 2009; Nuñez et al., 2009) and were among the earliest colonizers of isolated seedlings (Peay et al., 2012). In the greenhouse experiment, PR and PM seedlings were both well-colonized by *Suillus* spp. after 6 months. *Suillus* on PR have shown relatively higher activity with cellulases, hemicellulases, β-glucosidase, P- and N-containing organic compounds, and oxidative enzymes, which had a significant positive correlation with shoot biomass, root biomass, and phosphorus content of PR.

Ning et al. (2021) proposed that caution should be taken in the use of exotic hosts in the afforestation process because the potential shift of assembled EcMF community under exotic trees may alter below ground functional capabilities and symbiosis-mediated successional trajectories. However, after 2.5 years, we found the same EcMF community assemble patterns in our exotic and native pine hosts in the early stage, both PR and PM seedlings were well-colonized by *Suillus* sp. Meanwhile, in plantation, fungal and bacterial richness, diversity, and composition in rhizosphere or roots were not different between PR and PM (Supplementary Figures 4a,b). In our experiment, we could not detect any shift in the local EcMF community and host identity had no significant effect on EcMF communities. In addition, PR showed faster growth rate and higher survival rate than native PM. Therefore, here we suggest that PR is a suitable pine species for the restoration of degraded lands in Xifeng. However, we should be cautious about large-scale plantings of exotic PR plantations since mycorrhizal symbiosis of PR showed different enzyme activity and enzymatic functions. The needle nutrient concentration, C, P, and Fe of native PM are significantly higher than PR + LD and PR in the plantation. We assumed that the change in enzyme activity and enzymatic functions may have an effect on plant nutrient uptake. In addition, Wang et al. (2013) evaluated the impact of exotic and native trees species on soil nutrient availability and demonstrated that exotic species always had less P concentrations in leaves and stems than native species. Due to their faster growth rates, exotic plants would lower soil N or P availabilities than native species and lead to a nutrient limitation on plant growth. But exotics, with their fast growth rates, could retain more N in plant biomass and minimize leaching loss from the soil although they could not improve soil N availability in the short term. Hence, further study should be conducted for a better understanding of the relationship between host plant species and soil nutrient availability, then evaluating plantation succession and promoting forest restoration.

Moreover, Peay et al. (2011) proposed that in a non-invasive context, suilloid fungi often act as early successional species able to colonize pine seedlings, but they may be later displaced by late-successional fungi. Our current study was based on a 3-year-old plantation, so long-term monitoring is needed for a better understanding of the persistence of *Lactarius* and possible shifts of soil EcMF communities.

## Conclusion

This study analyzed the interaction effect of EcMF, including the co-introduced *L. deliciosus* and local EcMF communities, on the establishment of exotic *P. radiata* seedlings in Guizhou, Southwest China. The present results revealed that the introduced *P. radiata* seedlings could be successfully established in this new habitat with or without co-introducing *L*. *deliciosus*. The co-introduced *L. deliciosus* persisted in the Xifeng plantation after 3 years and was not replaced by the local EcMF. The high relative abundance of *L. deliciosus* had a positive correlation with the nitrogen acquisition of *P. radiata* and *L. deliciosus* pre-colonization significantly altered the mycorrhizosphere fungal composition. Some cosmopolitan species from *Suillus* genus are common in rhizosphere soil and root tips. A local EcMF, *Suillus* sp. in the local habitat formed a familiar association with *P. radiata* and we did not observe any shift of EcMF during the early establishment of *P. radiata*. Host identity had no effect on fungal community composition since *P. radiata* and native *P. massoniana* recruited similar EcMF communities. But the differences of enzyme activity and enzymatic functions of mycorrhizal symbiosis between *P. radiata* and *P. massoniana* may have an impact on plant nutrient uptake. Our study suggests that it is possible to establish exotic *P. radiata* plantations in Xifeng and achieve *L. deliciosus* fruiting, but a long-term monitoring is needed for a better understanding of the local fungal community changes induced by the *L. deliciosus* and the impact of enzyme activity and functions of ectomycorrhizas on plant nutrition uptake in the plantation.

## Data availability statement

The datasets presented in this study can be found in online repositories. The names of the repository/repositories and accession number(s) can be found in the article/Supplementary material.

## Author contributions

RW conducted the experiment, analyzed the data, and prepared the manuscript. YW conducted the experiment, analyzed the data, and contributed to the manuscript. AG-L contributed to fungal materials and revised the manuscript. PZ collected experimental materials and conducted the experiment. CC guided in the experiment plan and revised the manuscript. FY supervised the research and edited the manuscript. All authors contributed to the article and approved the submitted version.

## Footnotes

1
https://www.proseed.co.nz/catalogue/radiata-pine

2
https://unite.ut.ee
