# Supplementary material for: Factors influencing successful establishment of exotic Pinus radiata seedlings with co-introduced Lactarius deliciosus or local ectomycorrhizal fungal communities
Source: Front Microbiol. 2022 Nov 17;13:973483. doi: 10.3389/fmicb.2022.973483 (PMC9712797; doi:10.3389/fmicb.2022.973483)
Supplement: Supplementary file 1 [file Data_Sheet_1.docx]

**METHOD S1**: ECM fungal communication DNA extraction and ITS sequencing

Fungal DNA was extracted from 0.2 g of mycorrhizal root tips, root tips and mycorrhizospheric and rhizospheric soil using Powersoil™ DNA isolation kits (MoBio, San Diego, CA, USA) following manufacturer’s instructions for maximum DNA yield. The quality and quantity of the DNA extracts were checked using a spectrophotometer (Nanodrop, PeqLab, Germany). The ITS1 region was amplified using the forward primer ITS5 (5'- GGAAGTAAAAGTCGTAACAAGG-3') and the reverse primer ITS2 (5'- GCTGCGTTCTTCATCGATGC-3'). Purified amplicons were pooled in equimolar concentrations and pair-end sequenced on an Illumina MisSeq platform, Novaseq-PE250 (Personalbio®, Shanghai, China). The raw reads were analyzed using QIIME2 software (version 1.9.1, http://qiime2.org/) to trim off the low-quality reads, adaptors, barcodes, and primers. Sequences were clustered into operational taxonomic units (OTUs) by setting a 97 % similarity (Edgar 2010). The UNITE SH database v8.0 (http://unite.ut.ee/repository.php, Kõljalg et al., 2013) was used in chimera checking and OTU clustering. The Bray-Curtis distance-based dissimilarity distance, Simpson and Chao1 diversity index, principal coordinate analysis (PCoA) and a Venn diagram with shared and unique OTUs were performed on the Genescloud platform of Personalbio® to evaluate the fungal community differences between different samples. Raw sequence data have been deposited in the NCBI Sequence Read Archive database under the bioproject identifier PRJNA792310 and PRJNA 795335.

**REFERENCE**

Edgar RC. 2010. Search and clustering orders of magnitude faster than BLAST. Bioinformatics 26: 2460-2461.

Kõljalg U, Larsson KH, Abarenkov K, Nilsson RH, Alexander IJ, Eberhardt U, Erland S, Hoiland K, Kjoller R, Larsson E et al. 2005. UNITE: a database providing web-based methods for the molecular identification of ectomycorrhizal fungi. New Phytologist 166: 1063-1068.


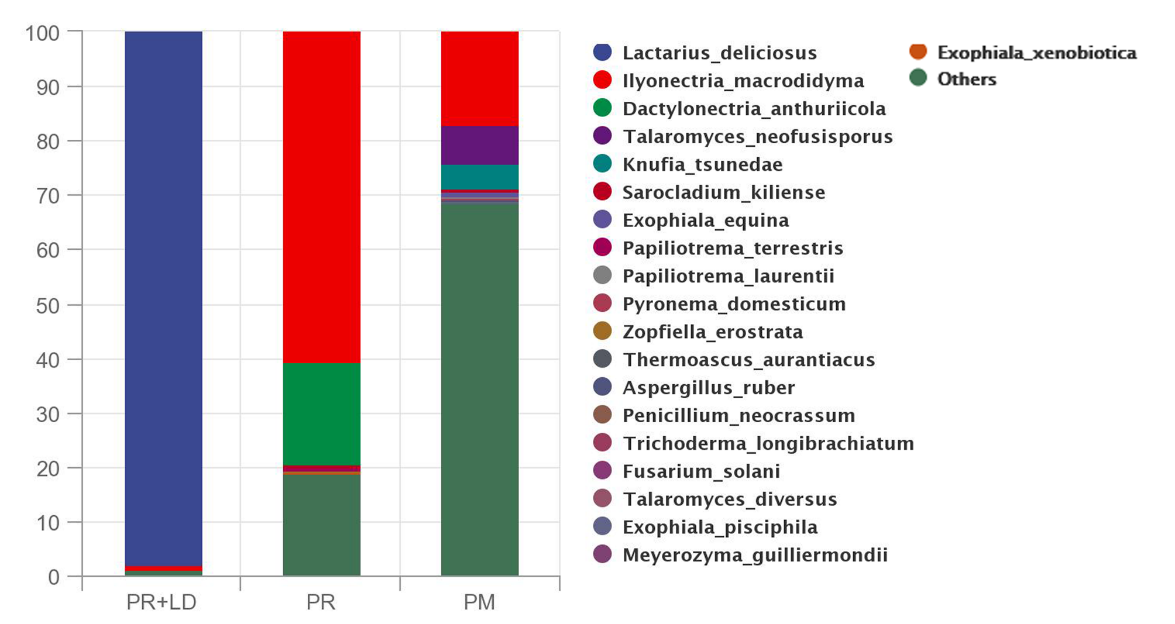


**FIGURE S1** Relative abundance of fungal community composition at the general level in root tips of *Pinus radiata* + *Lactarius deliciosus* (PR+LD), *P*. *radiata* (PR) and *P*. *massoniana* (PM) seedlings before plantation establishment.


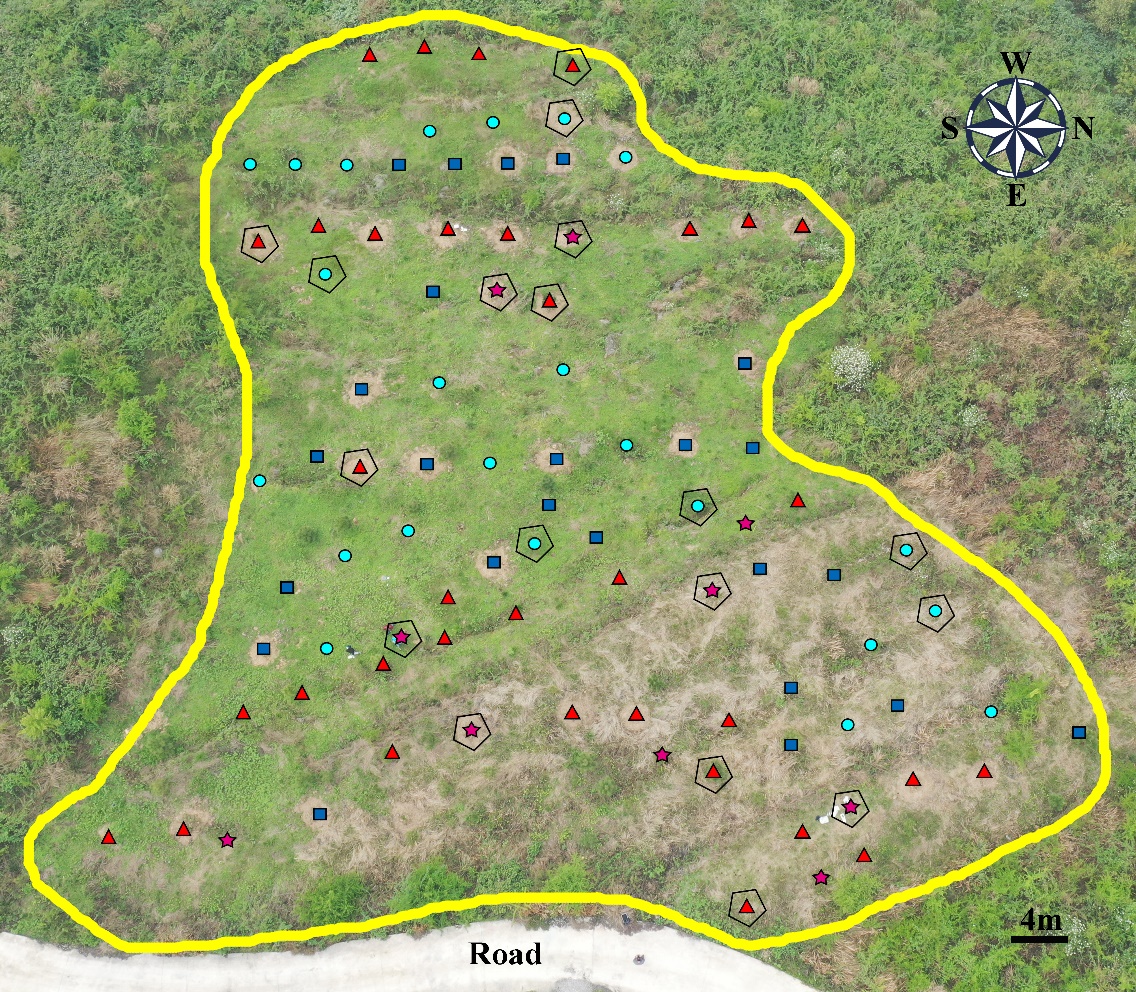


**FIGURE S2** Layout of plantation. Red triangles are PR+LD. Pink stars are PR. Light blue round circles are PM. Dark blue squares are PM+LV (*Lactarius vividus*, data not shown). Pentagons are samples were used for taking rhizosphere soil, roots and needles.


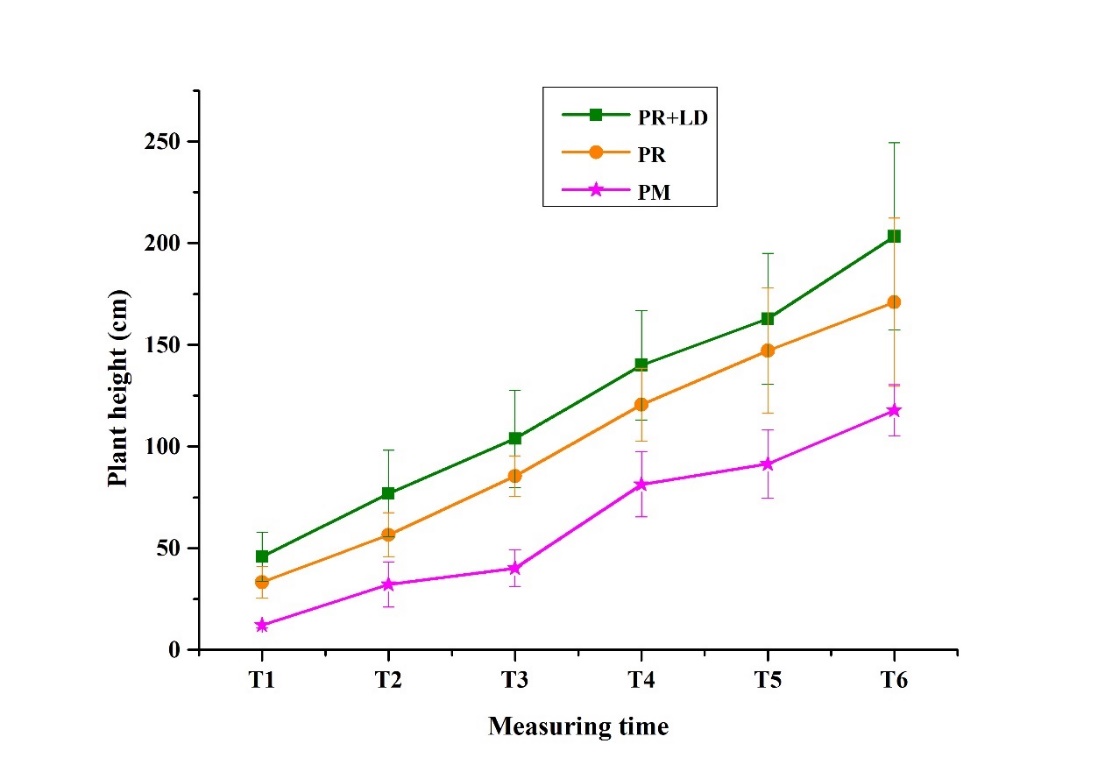


**FIGURE S3** Trees’ average height in every six months in Xifeng plantation. T1: Nov 2018, T2: May 2019, T3: Nov 2019, T4: May 2020, T5: Nov 2020, T6: May 2021.


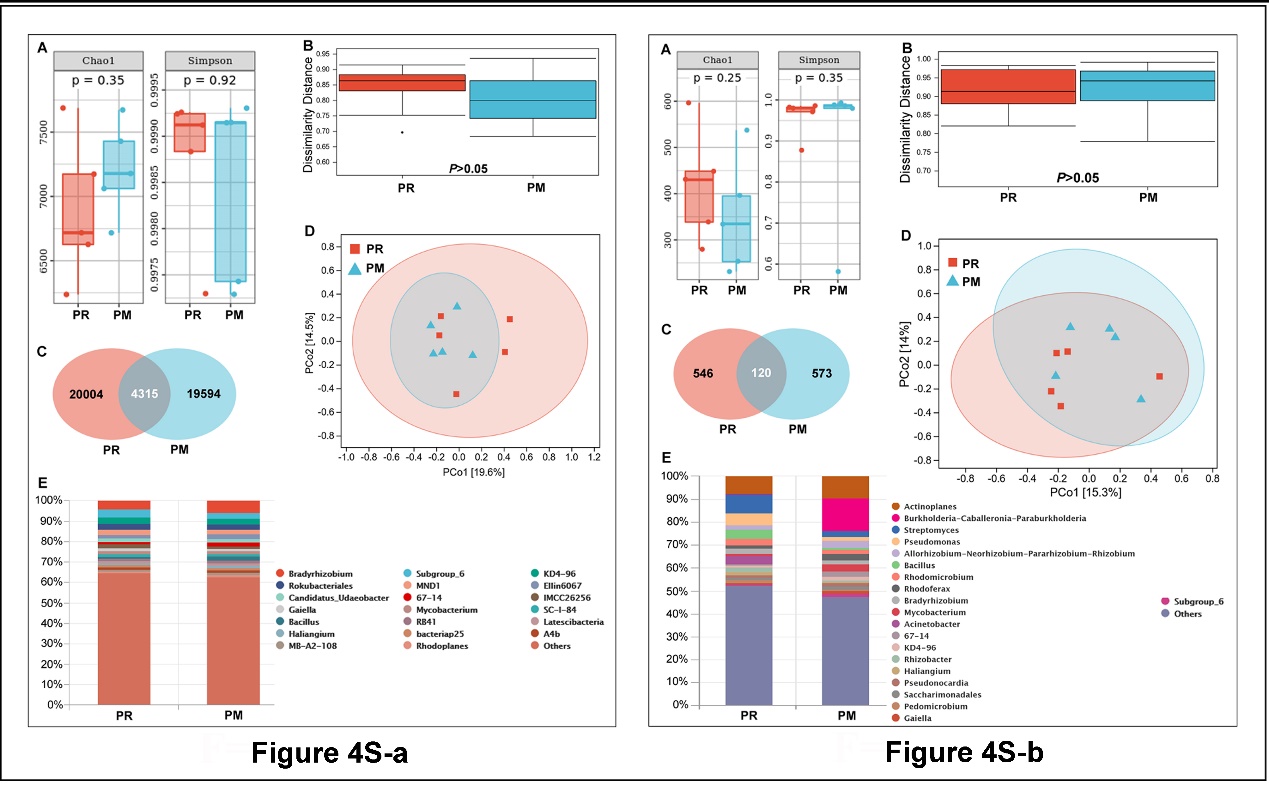


**FIGURE S4 a** Rhizosphere bacterial community of PR and PM trees in the Xifeng plantation. **b** Bacterial community of roots of PR and PM trees in the Xifeng plantation. (**A**) Chao1 and Simpson indexes. (**B**) Dissimilarity distance. (**C**) Venn figure showing shared and unique operational taxonomic units (OTUs) between samples of two trees. (**D**) Principal Coordinate analysis (PCoA). (**E**) Relative abundance of fungal composition at the genera level.

**TABLE S1** List of EcMF detected from mycorrhizosphere of *Pinus radiata* (PR+LD) and rhizosphere of *P. radiata* (PR) and *P. massoniana* (PM) in the Xifeng plantation, along with the presumed distribution and hosts.

| Speices | OTU_ID | Species hypothesis ID taxa | Host tree | Sampling area | Host trees |
| --- | --- | --- | --- | --- | --- |
| [*Amanita pseudovaginata*](https://unite.ut.ee/bl_forw.php?id=856253) | OTU_9129 | SH1611351.08FU | PR+LD, PR, PM | China, Pakistan | No information |
| *Geastrum triplex* | OTU­_640 | [SH1555997.08FU](https://unite.ut.ee/sh/SH1555997.08FU) | PM | Canada, Japan, Korea | No information |
| *Hygrocybe nigrescens* | OTU­_1179 | SH1512923.08FU | PR+LD, PR, PM | Canada | No information |
| *Lyophyllum decastes* | OTU­_2848 | [SH1588949.08FU](https://unite.ut.ee/sh/SH1588949.08FU) | PR | Asia, Europe, North America, South America, Oceania   \|  \|  \| \| --- \| --- \| | No information |
| *Russula brevipes* | OTU _12341 | [SH1509932.08FU](https://unite.ut.ee/sh/SH1509932.08FU) | PR+LD | China, Japan, Korea | No information |
| *Russula cerolens* | OTU_12131 | [SH1569811.08FU](https://unite.ut.ee/sh/SH1569811.08FU) | PR, PM | China | No information |
| *Russula compacta* | OTU_7715 | [SH1546768.08FU](https://unite.ut.ee/sh/SH1546768.08FU) | PM | Asia, Oceania | *Cymbidium*, *Lecanorchi* *Pinus* |
| *Russula cyanoxantha* | OTU_13484 | [SH1567120.08FU](https://unite.ut.ee/sh/SH1567120.08FU) | PM, PR | Asia, Europe, South America | *Carya*, *Corylus*, *Fagus*, *Picea*, *Pinus*, *Quercus* |
| [*Russula nauseosa*](https://unite.ut.ee/bl_forw.php?id=2957772) | OTU_3422 | [SH1625349.08FU](https://unite.ut.ee/sh/SH1625349.08FU) | PR+LD, PR, PM | Asia, Europe, North America, South America | *Alnus*, *Pinus*, *Pseudotsuga*, *Larix* |
| *Russula* sp1 | OTU_3845 | [SH1569729.08FU](https://unite.ut.ee/sh/SH1569729.08FU) | PM | Asia | *Cephalanthera* |
| *Russula* sp2 | OTU_7861 | [SH1633484.08FU](https://unite.ut.ee/sh/SH1633484.08FU) | PR | China, Thailand | *Aphyllorchis* |
| *Russula vesca* | OTU _1657 | [SH1633421.08FU](https://unite.ut.ee/sh/SH1633421.08FU) | PR+LD, PR | Asia, Europe, North America, Oceania | *Lecanorchis*, *Fagus*, *Pinus*, *Quercus* |
| *Scleroderma albidum* | OTU_9625 | [SH1544059.08FU](https://unite.ut.ee/sh/SH1544059.08FU) | PR+LD | Africa, Asia, Europe, North America, Oceania | *Eucalyptus*, *Salix* |
| *Scleroderma* sp. | OTU_11552 | [SH1526180.08FU](https://unite.ut.ee/sh/SH1526180.08FU) | PM | Asia, Europe, Oceania | *Castanopsis*, *Larix*, *Pinus*, *Populus*, *Quercus* |
| *Sistotrema brinkmannii* | OTU _4850 | SH1514167.08FU | PR | Africa, Antarctica, Asia, North America, South America, Oceania | *Pinus* |
| *Sistotrema coronilla* | OTU _10356 | SH1506095.08FU | PR+LD | Europe, North America, Oceania | *Quercus* |
| *Suillus collinitus* | OTU _5689 | SH1555181.08FU | PR+LD, PM | Africa, Asia, Europe, Oceania | *Pinus* |
| *Suillus placidus* | OTU _3575 | [SH1555178.08FU](https://unite.ut.ee/sh/SH1555178.08FU) | PR+LD, PR, PM | Africa, Asia, Europe, North America, Oceania   \|  \|  \| \| --- \| --- \| | *Pinus* |
| *Suillus* *pseudobrevipes* | OTU_9078 | [SH1555172.08FU](https://unite.ut.ee/sh/SH1555172.08FU) | PR, PM | Asia, Europe, North America, South America, Oceania | *Pinus* |
| *Tomentella pilosa* | OTU_4969 | [SH1528407.08FU](https://unite.ut.ee/sh/SH1528407.08FU) | PR | Asia, Europe, North America | *Castanea*, *Cistus*, *Fagus*, *Picea*, *Populus*, *Quercus*, *Tilia* |
| *Tuber indicum* | OTU_14388 | [SH1563426.08FU](https://unite.ut.ee/sh/SH1563426.08FU) | PR, PM | China, Spain | *Quercus* |
| *Tuber pseudohimalayense* | OTU_4250 | [SH1514135.08FU](https://unite.ut.ee/sh/SH1514135.08FU) | PM | China | No information |

**TABLE S2** OTUs shared by single root tips in identification and Illumina sequencing data in the greenhouse bioassay experiment.

| Root tips | Species | Best-fit of Illumina OTU | Best-fit of GenBank | SH | Species origin |
| --- | --- | --- | --- | --- | --- |
| PR | *Suillus* *placidus* | OTU_1487 | [MN258691](http://www.ncbi.nlm.nih.gov/nuccore/MN258691) | [SH1555178.08FU](https://unite.ut.ee/sh/SH1555178.08FU) | China |
| PM | *Suillus placidus* | OTU_4508 | KU721182 | [SH1555178.08FU](https://unite.ut.ee/sh/SH1555181.08FU) | China |

**TABLE S3** Eight enzyme activities of mycorrhizal root tips of two hosts in the greenhouse bioassay experiment.

|  | PR + *Suillus* (*n*=15) | PM + *Suillus* (*n*=15) |
| --- | --- | --- |
| C1^1^ (nmol mm^-2^ min^-1^) | 0.19 (0.00045) *** | 0.16 (0.0001) |
| GC (pmol mm^-2^ min^-1^) | 26.95 (0.009) *** | 21.19 (0.93) |
| GD (pmol mm^-2^ min^-1^) | 4.03 (1.22) * | 1.88 (0.1) |
| LAC (pmol mm^-2^ min^-1^) | 15.43 (1.77) ** | 7.72 (0.02) |
| X (pmol mm^-2^ min^-1^) | 12.2 (0.076) | 13.02 (0.72) |
| LEU (pmol mm^-2^ min^-1^) | 49.41 (1.86) *** | 32.06 (0.9) |
| NAG (pmol mm^-2^ min^-1^) | 14.59 (1.06) | 13.08 (0.59) |
| ACP (nmol mm^-2^ min^-1^) | 0.37 (0.02) ** | 0.3 (0.02) |

^1^C1−cellobiohydrolase, GC−β-glucosidase, GD–β-glucuronidase, LAC–laccase, X–β-xylosidase, LEU–leucine aminopeptidase, NAG–N-acetylglucosaminidase, ACP – acid phosphatase, *P* = significance, * *P* < 0.05; ** *P* < 0.01; *** *P* < 0.001.

**TABLE S4** The Pearson correlation analysis between eight enzymes and seedling vigor status (*Pinus radiata* seedling N = 15, *P. massoniana* seedling N = 15. *P* = significance (<0.05 in bold).

| Host | Variable | C1 | GC | GD | LAC | X | LEU | NAG | ACP |
| --- | --- | --- | --- | --- | --- | --- | --- | --- | --- |
| PR | Shoot biomass | -0.542  ***P*=0.037** | 0.041  *P*=0.885 | -0.592  ***P*=0.02** | 0.523  ***P*=0.045** | 0.294  *P*=0.288 | 0.192  *P*=0.439 | 0.643  ***P*=0.01** | 0.631  ***P*=0.012** |
|  | Root biomass | -0.347  *P*=0.205 | -0.207  *P*=0.46 | -0.107  *P*=0.705 | 0.215  *P*=0.441 | 0.435  *P*=0.105 | 0.622  ***P*=0.013** | 0.007  *P*=0.979 | 0.007  *P*=0.982 |
|  | C | 0.264  *P*=0.342 | 0.007  *P*=0.98 | 0.044  *P*=0.876 | -0.009  *P*=0.976 | -0.197  *P*=0.483 | -0.065  *P*=0.817 | -0.207  *P*=0.459 | -0.146  *P*=0.603 |
|  | N | 0.352  *P*=0.198 | 0.286  *P*=0.301 | 0.201  *P*=0.472 | -0.265  *P*=0.339 | -0.390  *P*=0.151 | -0.583  ***P*=0.022** | -0.069  *P*=0.808 | -0.023  *P*=0.934 |
|  | P | 0.221  *P*=0.429 | 0.194  P=0.489 | 0.53  ***P*=0.042** | -0.306  *P*=0.267 | -0.274  *P*=0.322 | -0.417  *P*=0.122 | 0.137  *P*=0.628 | 0.148  *P*=0.599 |
| PM | Shoot biomass | -0.037  *P*=0.896 | -0.274  *P*=0.324 | -0.415  *P*=0.124 | -0.449  *P*=0.093 | -0.23  *P*=0.41 | -0.067  *P*=0.812 | 0.117  *P*=0.677 | 0.083  *P*=0.769 |
|  | Root biomass | -0.037  *P*=0.997 | 0.108  *P*=0.702 | 0.186  *P*=0.507 | 0.131  *P*=0.643 | 0.149  *P*=0.595 | 0.461  *P*=0.084 | -0.129  *P*=0.646 | 0.234  *P*=0.401 |
|  | C | -0.172  *P*=0.54 | 0.209  *P*=0.454 | 0.374  *P*=0.17 | -0.078  *P*=0.782 | 0.586  ***P*=0.022** | -0.041  *P*=0.886 | -0.594  ***P*=0.019** | -0.254  *P*=0.361 |
|  | N | 0.209  *P*=0.455 | 0.013  *P*=0.964 | -0.209  *P*=0.456 | -0.015  *P*=0.956 | 0.243  *P*=0.382 | -0.04  *P*=0.887 | -0.08  *P*=0.777 | 0.247  *P*=0.375 |
|  | P | -0.388  *P*=0.154 | 0.26  *P*=0.35 | 0.334  *P*=0.224 | -0.364  *P*=0.182 | -0.088  *P*=0.755 | 0.017  *P*=0.952 | -0.067  *P*=0.811 | 0.096  *P*=0.733 |

|  | |  | |  | |  |
| --- | --- | --- | --- | --- | --- | --- |
|  |  | |  | |  |  |
